# Brain Tissue Cysts in Infected Mice with RH-Strain of *Toxoplasma gondii* and Evaluation of BAG1 and SAG1 Genes Expression

**Published:** 2013

**Authors:** Monavar Selseleh, MH Modarressi, S Shojaee, M Mohebali, MR Eshraghian, Mina Selseleh, H Keshavarz

**Affiliations:** 1 Dept. of Medical Parasitology and Mycology, School of Public Health, Tehran University of Medical Sciences, Tehran, Iran; 2 Dept. of Genetic, School of Medicine, Tehran University of Medical Sciences, Tehran, Iran; 3 Center for Research of Endemic Parasites of Iran (CREPI), Tehran University of Medical Sciences, Tehran, Iran; 4 Dept. of Epidemiology and Biostatistics, School of Public Health, Tehran University of Medical Sciences, Tehran, Iran

**Keywords:** *Toxoplasma gondii*, RH strain, RT-PCR, SAG1, BAG1

## Abstract

**Background:**

*Toxoplasma gondii* is an obligate intracellular parasite that infects humans at high prevalence rates. The virulent RH strain of *T. gondii* is generally considered to have lost its cyst forming capacity. This study performed to obtain tissue cysts in mice infected with tachyzoites of RH strain treated with sulfadiazine (SDZ). It provides the opportunity to analyze the conversion of tachyzoite to bradyzoite stage of the RH strain, followed by stage-specific gene-expression analyzing.

**Methods:**

Two groups of Swiss-Webster and BALB/c mice were infected subcutaneously with 10^4^ tachyzoites of *T. gondii*, RH strain and given SDZ (300 mg/l) with NaHCO3 (5 g^-1^) in drinking water from day1 to day 14 post infection (p.i). The infected mice were sacrificed on day 50 post infection. Their brains were removed and the numbers of tissue cysts were microscopically counted. Total RNA was extracted from brains and cDNA synthesis was carried out. Finally, RT-PCR (Reverse transcription PCR) was used to detect the expression of bradyzoite (BAG_1_) and tachyzoite (SAG_1_) specific genes during tachyzoite / bradyzoite stage conversion.

**Results:**

Sixty five percent of all infected mice were survived. Cysts were detectable in mice brain (45%) on day 50 p.i. Also RT-PCR of the brain samples was positive for SAG1 and BAG1.

**Conclusion:**

It seems that conversion of tachyzoites to bradyzoites in brain of mice undergoing SDZ was not completed until 50 days after inoculation.

## Introduction

Toxoplasmosis is a worldwide disease caused by a protozoan parasite, *Toxoplasma gondii* (1). Although infection with this parasite is typically asymptomatic in healthy individual, *T. gondii* causes substantial morbidity and mortality in immunocompromised patients and in congenitally infected infants (2, 3).

The virulence of *T. gondii* strains is commonly detected according to the outcome of infection in mice (4, 5). Strains that induce mortality are considered virulent and conversely, those inducing chronic infection are considered avirulant. Studies of the population genetic structure have shown that all *T. gondii* strains belong to three clonal lineages, of which type 1 includes mouse virulent strains, and type 2 and 3, were mouse avirulant (6, 7).

One of the typical type1 is the RH strain which was first isolated by Sabin in 1939 (8). The RH strain has lost its capacity to induce oocyst formation in cats (9), while tissue cysts have been developed in non-immune wild-type mice exclusively after early treatment with sulfadiazine (SDZ). Addition of SDZ to the drinking water of infected mice could significantly reduce the parasite burden (10).

Key to *T. gondii* pathogenesis is its ability to differentiate from a rapidly replicant, tachyzoite stage to a relatively non-immunogenic and dormant bradyzoite stage in tissue cyst. These bradyzoites can reconvert back to tachyzoites years later and causing serious pathological symptom and death if a person becomes immune-compromised (2). Stage conversion between tachyzoite and bradyzoites not only plays an important role in establishing a chronic infection, but also it is responsible for disease reactivation (3). Understanding of this process could also help in designing new chemotherapeutic agents capable of eliminating tissue cyst, yet poorly understood.

“Since tachyzoites and bradyzoites are alike in their structures and cannot be discriminated under a light microscope, some techniques are used to detect *Toxoplasma* tachyzoite-bradyzoite stage conversion, for example electron microscopy, micro array, immunohistochemistry and lately transfection with fluorescent proteins (11)”. Comparison with these techniques, RT-PCR method has the advantages of being fast which needs only basic PCR equipment. Therefore it offers as useful tool for studying and diagnosis the tachyzoite and bradyzoite interconversion process.

Expression of tachyzoite- specific genes is switched off and bradyzoite- specific genes starts to be up- regulated in the early process stage conversion (12). Many stage specific genes of *T. gondii* have been detected with the development of molecular biological technologies. Classification is based on the localization and the function of the gene products. The parasite's cell surface is covered by a family of developmentally regulated, glycosyl phosphatidyl inositol-linked surface proteins, named SAG (13). Among these, surface antigen one (SAG_1_) is a well known tachyzoite- specific gene with complete expression profile. SAG_1_ is likely play an important role in parasite attachment, penetration into the host cell and immune modulation (14, 15). Bradyzoite antigen (BAG1) is the most abundant bradyzoite specific gene (16, 17). BAG1 expression is up- regulated early in the differentiation process. It can be found in the cytoplasm and has homology with small heat Shock proteins in plants (18).

Enolase (ENO1) and lactate dehydrogenase (LDH2) are other enzymes found in cytoplasm of bradyzoites only. ENO1 and LDH2 mRNAs seems to be presented only in the bradyzoite stage. However, because ENO1 and ENO2 shared 73.6% of amino acid sequences similar to LDH1 shared 71.4% with LDH2, made these primers less potent stage-differentiating RT-PCR (19). *T. gondii* deoxyribose phosphate aldolase- like gene (TgDPA) with 31 kDa coding with 286 amino acids has been sequenced and was highly expressed in bradyzoite only (16), however, the function of TgDPA is still unknown. The matrix antigen (MAG1) was initially identified as a 65 kDa protein expressed within the cyst and in the cyst wall surrounding the bradyzoites, however, there was a study demonstrated that MAG1 is also expressed in tachyzoites (20).

The aim of this study was analyzing RT-PCR assay based on the expression of the stage- specific genes SAG1-tachyzoites and BAG1-bradyzoites for detecting early- stage conversion in *Toxoplasma gondii* RH strain in animal model.

## Materials and Methods

### Animals and parasites

A total of 25 male mice aged 5- 6 weeks with an average weight of 18- 20 g selected (5 Swiss Webster and 20 BALB/c). Tachyzoites of *T. gondii* RH strain maintained through serial intra-peritoneal (i.p.) passage were used for experimental infection. Tachyzoites were harvested from mouse peritoneal cavity 72 h post infection (p.i.), the parasites were counted and adjusted to 10^4^/ ml in saline. Each 0.5 ml solution was inoculated subcutaneously to each mouse.

### Experimental design

Briefly, groups of mice were inoculated subcutaneously with 10^4^ tachyzoite of *T. gondii*, RH strain. Drug SDZ was administrated at 300 mgl^-1^ adding NaHCO3 5gl^-1^ in drinking water.

SDZ treatment was initiated 24 h p.i and continued for 14 days. The control group consists of mice inoculated subcutaneously (s.c.) with 10^4^ tachyzoites of *T. gondii*, RH strain without treatment with SDZ.

### Counting of tissue cysts

The infected mice were sacrificed on day 50, their brains were removed and homogenized with 1ml of phosphate buffer saline (P.B.S, pH = 7.2) by passing through 23 gauge needle. The number of tissue cysts was determined by placing 2 drops of each 20 µl brain homogenate on slides and counted under a light microscopy (21). The number of cysts per brain was calculated by the number of counted cysts in 2 drops multiplying in 25.

### RNA extraction

Total RNA was extracted using Tripure reagent according to manufacture procedure (Roche, Germany). Briefly, 80-100 mg of brain tissue were cut into small pieces and homogenized by squeezing through 20 gauge needle in 1ml of Tripure reagent. The RNA concentration was determined using a spectrophotometer, followed by denaturing gel electrophoresis to reveal RNA integrity.

### cDNA synthesis and RT- PCR

cDNA synthesis was carried out using Quantitect reverse transcription kit (Fermentase) with some minor modification procedure. The mixture of 1µg RNA, 2 µl gDNA Wipeout buffer and 11µ RNAse- free water was incubated at 42 °C for 2 min. One microliter of Quantiscript reverse transcriptase, 4µl of RT buffer and 1µl of RT primer were added and incubated for 15 min at 42 °C and 3 min at 95 °C to inactivate the enzyme. Three pairs of primers for the SAG_1_ and BAG_1_ and beta- actin (as internal control) designed using National Center for Biotechnology Information site (Table 1).

**Table 1:** Specific primers set for genes

Gene	Primer	T. anneling^.c^
SAG1	S 5'GCTGTAACATTGAGCTCCTTGASTTCCTG3’ AS 5'CCGGAACAGTACTGATTGTTGTCTTGAG3’	58.5
BAG1	S 5'AGTCGACAACGGAGCCATCGTTATC3’ AS 5'ACCTTGATCGTGACACGTAGAACGC3’	57
Hose keeping (beta actin)	S5'GACCTTACCGAGTACATGATGAAG3’ AS 5'CCATCGGGCAATTCATAGGAC3’	58

The procedure of RT- PCR was carried out using 25 µl RT- PCR reactions containing Taq polymerase 1 µl, MgCl_2_ 1 µl, dNTP 2 µl, SAG_1_ primers 2.5 µl, BAG_1_ primers 2.5 µl, beta actin primer 2.5 µl, cDNA 2 µl and H_2_O 11.5 µl. An initial denaturation step at 94 °C for 5 min, then 40 cycles consisting of 94 °C for 30 sec, 62 °C for 30 sec, 72 °C for 30 sec and a final extension step at 72 °C for 10 min. The RT-PCR products (10µl) were finally separated and visualized on 1.8% of agarose gel. Meanwhile, No-RT control was used to detect genomica DNA contamination in samples. Production of No-RT control is the same as cDNA procedure but it does not have reverse transcriptase enzyme. Consequently, we must not observe any band with this sample after PCR and observation any band in this sample shows contamination RNA with genomic DNA.

This study was reviewed by Ethical Committee of Tehran University of Medical Sciences and accepted in project no: 240/2503.

## Results

### Effects of SDZ treatment

**Fig. 1:**
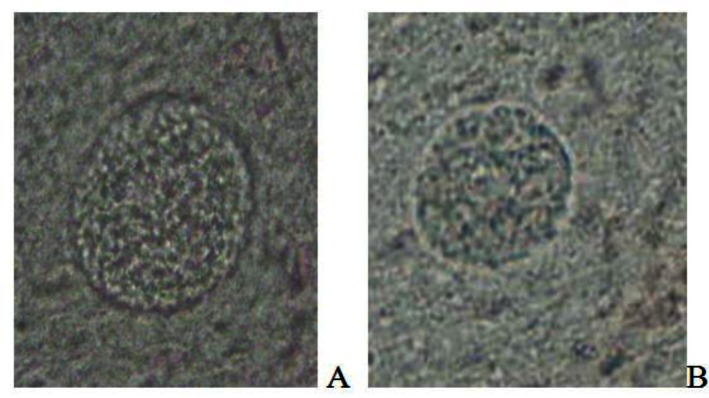
A, B Optical microscopic appearance of RH strain cysts in mouse- brain tissue (40X)

The rate of infected mice treated with SDZ, was compared with control group. All the mice in control group died between days 4 and 8 p.i. All the mice treated with SDZ became ill (reduced locomotion and ruffled hair) from day 4 to day 9 p.i. Sixty five percent of the subcutaneously infected and SDZ-treated mice were survived and in 45% of mice brain tissue cysts were found (Fig. 1).

Tissue cysts in all of Swiss Webster mice (n = 5) and in some of BALB/c mice (n = 7) were observed. The number of tissue cysts in the brains was almost 140 ± 10 cysts in days 50 p.i. However cysts were not seen in brain samples of day 30th.

### RT-PCR

**Fig. 2:**
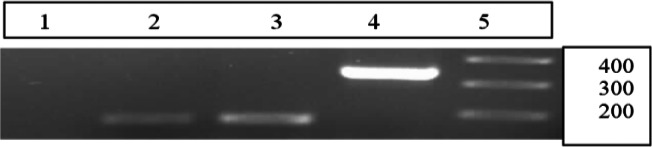
Bradyzoite and tachyzoite detection by RT-PCR targeting *T. gondii* BAG1 and SAG1 and beta actin genes in samples of brain. Lanes are shown as 1: no RTase, 2: beta-Actin, 3: BAG1 (cDNA), 4: SAG1 ( cDNA) and 5: Size Marker

For detecting early-stage conversion of *T. gondii* RH strain, RT-PCR was carried out to show the expression of the stage specific genes SAG_1_ and BAG_1_ which are specific for tachyzoites and bradyzoites, respectively. The expected size of the amplified gene products in the RT-PCR for SAG_1_ was 350 bp and for BAG_1_ was 200 bp (Fig. 2).

Results of RT-PCR showed that SAG1 and BAG1 genes were expressed in the brain samples of mice fifty days post infection. However BAG1 transcript was not present in the brain samples of day 30th. There is a size difference using BAG1 primers for cDNA versus genomic DNA based on designing primers.

**Fig. 3:**
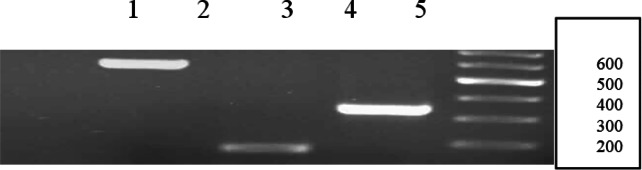
Amplification products from bradyzoite and tachyzoite stages of *T.gondii using genomic DNA*. Lanes are shown as 1: no RTase, 2: BAG1 (g DNA), 3: beta-Actin,4: SAG1(g DNA)

RT-PCR for BAG1 had 200 bp band, while PCR for BAG1 with genomic DNA had 635 bp band. There was no difference between results of RT-PCR (cDNA) and PCR for SAG1 (DNA), both of them had 200 bp bands. If we see a 200 bp band in No-RT (negative control), it shows that RNA is contaminated with genomic DNA (Fig. 3).In present study, No-RT control did not show any band.

## Discussion

The RH strain of *T. gondii* belongs to type one (6). Cysts of the type one strains cannot be produced in non-immune and non-treated mice (5). The cyst form is an important form in the life cycle of the parasite concerning its pathogenesis in immunocompromised patients and the ability for development of *Toxoplasma* encephalitis. Chronic infection models are suitable for evaluating of drugs efficacy against the cyst form of *T. gondii*.

There are several reports of obtaining *Toxoplasma gondii* RH strain cyst in vivo and in vitro conditions. In vitro models of the tachyzoite–bradyzoite differentiation have been demonstrated using a variety of stress conditions including treatment of host cells with gamma interferon or mitochondrial inhibitors (22), or by employing alkaline pH or high temperature (23). The use of artificial stress conditions, for example alkaline pH, may be effective method for processing of *Toxoplasma* stage conversion, nevertheless, we have to admit that it may not mimic those conditions need for stage conversion during the natural course of infection. Therefore, we used animal model for obtaining tissue cyst of *T. gondii* RH strain.

In this study a model for RH cyst production in mice treated with SDZ was described. This drug may inhibit the propagation of fast growing parasites; as a result tachyzoites could be differentiated into bradyzoites. The success of s.c. injection is resulted by slowing spread of the parasite allowing enough time for conversion to bradyzoite. In this research, sixty five percent of the subcutaneously infected and SDZ-treated mice survived, and in 45% of mice tissue cysts were found in brain. The number of tissue cysts in the brains was almost 140 ± 10 cysts in 50 days post infection. It is in accordance with before research (10). Our result showed that, BALB/c mice were suitable for obtaining cyst as well, in spite of the fact that it seems the Swiss Webster mice are more susceptible than BALB/c for producing cysts of *T. gondii*, RH strain. Because of tissue cysts were observed in all of Swiss Webster mice (n = 5) and in some of BALB/c (n = 12). It should be noted that in treated mice, the cysts varied from the viewpoint of size and quality, and both large and small cysts contained numerous bradyzoites.

In different experience, we tried to obtain RH strain cysts in Wistar rats. Two groups of Wistar rats were inoculated with 10^6^ tachyzoite (n = 8), 10^7^ tachyzoite (n = 10). 8 weeks post infection, we couldn't obtain tissue cysts in brains of rats, and it was confirmed with bioassay test too. It is in contrast with before studies, that they were successful in obtaining RH strain cysts in Fischer rats (15, 24). It seems that the strain of rat is important for obtaining tissue cyst. This hypothesis need to more studies for conformation. In mice, RH cysts have been produced after immunization by *T. gondii* lysate (25) but this model was not proper for subsequent immunological studies. RH cysts could also be produced in mice by treatment with 125 mg/day of SDZ among 1 and 21 days p.i, this treatment reduced mortality of mice and produced easily recognized cysts (26).

Stage conversion between tachyzoite and bradyzoite forms is associated with morphologic, molecular and biological changes, including stage specific antigen expression. There are different ways for evaluating genes expression for example micro array, RT-PCR and real –time (27, 28). Efficacy of RT-PCR for detecting *T. gondii* bradyzoite gene expression in CSF samples from AIDS patients was proved by Cultrera et al. (29).They showed that MAG1 and SAG4 genes were specific to bradyzoite stage in TE relapse patients. In our study, BAG1 seems to have enough expression level for discrimination of bradyzoite stage. Therefore, we selected BAG1 as marker to determine bradyzoite stage by RT-PCR. The results showed that BAG1 and SAG1 genes were expressed in the brain samples of day 50th. This finding proved that conversion tachyzoite to bradyzoite has not completed in this period and complete conversion to bradyzoite probably takes more time.

“PCR assays using gDNA as a template have been unable to discriminate between an increase or decrease in SAG1 and BAG1 expression between the active tachyzoite stage and the latent bradyzoite stage” (30). Our RT-PCR used cDNA as a template; it was certainly able to amplify both stage- specific genes with different expression levels. SAG1 and BAG1 mRNAs have hardly identified by conventional hybridization methods like Northern analysis (19), but these techniques need large amounts of target RNA.

## Conclusion

Our results indicates that treatment with SDZ is an effective way for producing tissue cysts of *T. gondii*, RH strain in mice(Swiss Webster mice and BALB/c), also RT-PCR is a powerful and fast method for detecting *Toxoplasma* tachyzoite -bradyzoite stage conversion. This method would be useful for in vivo drug efficacy studies, where it is important to detect parasite stages, for designing a new drug to eliminate tissue cysts. This RT-PCR technique can be used to clinical specimens of immuno-compromised patients (HIV patients or transplant patients) for the early identification of tachyzoite- bradyzoite stage conversion.
